# Preliminary Research of Relationship between Acute Peritonitis and Celiac Primo Vessels

**DOI:** 10.1155/2013/569161

**Published:** 2013-08-28

**Authors:** Xiaoyu Wang, Hong Shi, Jingjing Cui, Wanzhu Bai, Wei He, Hongyan Shang, Yangshuai Su, Juanjuan Xin, Xianghong Jing, Bing Zhu

**Affiliations:** ^1^Institute of Acupuncture and Moxibustion, China Academy of Chinese Medical Sciences, Beijing 100700, China; ^2^Shandong University of Traditional Chinese Medicine, Jinan 250355, China

## Abstract

Previous studies demonstrated that primo vessels (PVs) were distributed in different parts of the body in mammals, and PVs were also involved in some processes of pathology such as cancer. Whether PVs are intrinsic structures in mammals or not is still ignored. In this study, a peritonitis model rat was induced by i.p. administration of *E. coli* in rats. PVs were observed in all infected rats, but it appeared less in untreated rats, taking 10.53% (4/38). In addition, we examined cell types in celiac PVs by fluorescent staining with 4′,6-diamidino-2-phenylindole (DAPI) and Alexa Fluor 488 phalloidin, as well as immunofluorescent staining with CD11b and intercellular adhesion molecule-1(ICAM-1), and found the following. (1) The rod-shaped nuclei aligned longitudinally along PVs. (2) DAPI-, phalloidin-, CD11b-, and ICAM-1-positive labeling coexisted in PVs, suggesting that fibroblasts and leucocytes might be two kinds of cell types in PVs for both infected and control rats. (3) The difference was that numerous cells in PVs of the infected rats contained DAPI-labeled multilobal nucleus and were expressed with CD11b- and ICAM-1-positive labeling on the cytoplasm and membrane, showing the typical characteristics of neutrophil. (4) The cells in PVs from the untreated rats are those of loose connective tissue. Therefore, it is reasonably considered that PVs from infected rats might be the pathological products which might be involved in inflammation.

## 1. Introduction

Bong-han Kim reported for the first time in 1962 that the Bonham system, which was considered as the anatomical basis of classical acupuncture meridians, included several subsystems such as Bonghan corpuscles and Bonghan ducts [1, 2]. The structure was also found by Fujiwara's follow-up [3]. Unfortunately, Bonghan theory was not clearly confirmed by most investigators [4] because the method employed by Kim was not disclosed and the experiments were hard to reproduce.

Recently, a series of reports Professor Soh KS's group showed that the primo vessels (PVs), which were referred to Bonghan ducts (BHDs) and identified as a part of a circulatory system by Kim [1, 2] in the early 1960s, could be found in different parts of the body such as on the surface of the internal organs of rats, rabbits, and swine [5–7], inside the blood and lymphatic vessels [8, 9], in the epineurium, running along the sciatic nerve [10], and below the skin [11]. However, whether PVs are intrinsic structures in mammals or not still remains elusive. 

In order to confirm the structure of PVs, a lot of labs in Korea, China, and USA tried to repeat the labeling methods of PVs provided by Soh. According to the method reported by Lee et al. [12], PVs in enterocoelia were identified and stained by dropping 0.2% diluted Trypan blue solution. But PVs can not be found in all the subjects by the staining method. During experiments of searching PVs we found that the percentage of celiac PVs emergence was related to a lot of factors such as age and method of anesthesia. This indicated that the PVs may be related to a pathological process. Recent studies showed that PVs played an important role in cancer, especially in tumor metastasis, and regeneration [13, 14]. Here a question was raised: is PV an intrinsic structure of mammals or pathological products? In this study, we tried to address the relationship between peritonitis and celiac PVs by establishing an acute peritonitis model. We first summed up the data during the search of PVs in juvenile rats and adult rats, compared the different emergence of PVs due to different methods of anesthesia. Moreover, whether acute peritonitis correlated with the occurrence of celiac PVs was investigated by fluorescent staining with 4′,6-diamidino-2-phenylindole (DAPI) and Alexa Fluor 488 phalloidin, as well as immunofluorescent staining with CD11b and intercellular adhesion molecule-1(ICAM-1). The results will be helpful to understand the cell types and chemical characteristics in PVs. 

## 2. Materials and Methods

### 2.1. Animal Preparation

Male and female Sprague-Dawley (SD) rats weighing 150–350 g were purchased from Institute of Animal, Academy of Chinese Medical Sciences. The animals were housed under a 12 h light/dark with free access to food and water. All animals were treated according to the Guide for Use and Care of Medical Laboratory Animals from Ministry of Public Health of People's Republic of China. 

### 2.2. Animal Model of Acute Peritonitis and Cytometry of Blood and Reroperitoneum

Fifity-nine male and female SD rats, weighing 250–350 g were conducted in the experiments. 39 rats were subjected to acute peritonitis and 20 rats as control. Acute peritonitis model (PM) was made through intraperitoneal (i.p.) injection of sterile phosphate-buffered saline (PBS, 10 mL/kg) containing 3.3 × 10^8^ colony forming units of *E. coli* (ATCC 25922, Beijing Century Ocote Biotechnology Co. Ltd.) [15]. Control animals just received the same volume PBS.

24 h after i.p. injection of* E. coli*, the rats were anesthetized by intramuscular injection (i.m.) of 10% urethane (1.5 g/kg). The reroperitoneum of the rats was collected for leukocyte counting. The venous blood was collected for blood analysis. Cell counting was performed by automated blood cell analyzer (Nihon Kohden, 5108K). 

### 2.3. Methods for Identifying PVs

To identify PVs inside the abdominal cavity, surgery was performed in rats. All surgical procedures were performed under anesthesia with urethane (1.5 g/kg^−1^) i.p. or i.m. For acute peritonitis and control, the operation was done 24 h after i.p. injection of *E. coli* and PBS. The middle of the rats' abdomen was incised and the intra-abdominal organs were exposed carefully. Then a 0.2% trypan blue solution 1-2 mL was dropped on the exposed organs as in the previous report [12]. After rinsing away the dye with warm saline, primo nodes and primo vessels were identified through a surgical microscope (SZX12, Olympus, Japan). Finally, the images were captured with a CCD camera (Nikon SMZ750) *in situ* and *in vivo*.

### 2.4. Fluorescent Staining of PVs with Alexa Fluor 488 Phalloidin and DAPI

After intraoperative imaging of the PVs, *in vitro* examination was performed with histological staining of the samples. PVs were fixed with 4% paraformaldehyde in 0.1 M phosphate-buffered solution (PB, pH 7.4) for 2 hours at 4°C, then moved to 25% sucrose in 0.1 M PB (pH 7.4). Alexa Fluor 488 phalloidin (specific to F-actin) and DAPI were employed to identify the morphology of cell nuclei and F-actin, and the cells shape and arrangements of PVs were clearly detected. The PVs were incubated for 2 h by Alexa Fluor 488 Phalloidin, which was dissolved in methanol (1 : 50; Molecular Probes, Eugene, OR, USA) in the dark at room temperature (RT) and then washed with 0.1 M PB. DAPI (0.1 mg/mL, Molecular Probes, Eugene, OR, USA) was added and incubated for 30 min. 

### 2.5. Fluorescent/Immunofluorescent Staining with DAPI, Alexa Fluor 488 Phalloidin, CD11b and CD54/ICAM-1

Fluorescent staining with DAPI and Alexa Fluor 488 Phalloidin were used for identifying the morphology of cell nuclei and F-actin, while immunofluorescent staining with CD11b and CD54/ICAM-1 was used for determining the immune cells and intercellular adhesion molecule that was mainly expressed in the membrane of neutrophils. The staining included DAPI/Phalloidin, DAPI/CD11b, and DAPI/ICAM-1.

Before staining, the PVs mounted on the microslide were incubated in a blocking solution containing 3% normal goat serum and 0.5% Triton X-100 in 0.1 M phosphate-buffered solution (PB, pH 7.4) for 30 min. For phalloidin staining, the sample was further stained with Alexa Fluor 488 phalloidin dissolution (1 : 50; Molecular Probes, Eugene, OR, USA) for 2 h and then washed with 0.1 M PB. After that, the tissue was counterstaining with DAPI (Molecular Probes, Eugene, OR, USA). For CD11b staining, the sample was further transferred to mouse anti-CD11b monoclonal antibody (1 : 1000; Abcam, Hong Kong) at a dilution of 0.1 M PB containing 0.5% Triton X-100 overnight at 4°C. On the following day, after washing three times with 0.1 M PB, PVs were exposed to Alexa Fluor 594 goat antimouse IgG secondary antibody (1 : 500; Molecular Probes, Eugene, OR, USA) for 2 h and then washed with 0.1 M PB. After that, the tissue was counterstaining with DAPI. The procedure for ICAM-1 staining was the same as for CD11b staining. The difference is that rabbit anti-ICAM-1 antibody was used as primary antibody (1 : 100; Bioss, China) and Alexa Fluor 488 goat antirabbit IgG as secondary antibody.

### 2.6. Observation and Statistical Analysis

All Quantitative data are expressed as mean ± SE. The tissue samples were observed and recorded with confocal imaging system (FV1000, Olympus, Japan) and analyzed by the Olympus Image Processing Software. Comparisons between two groups were analyzed by independent *t* test. *P* < 0.05 was considered as a statistical significance. 

## 3. Results

### 3.1. Intraoperative Visualization of PVs and the Related Factors of Emergence Rate of Celiac PVs

**Figure 1:**
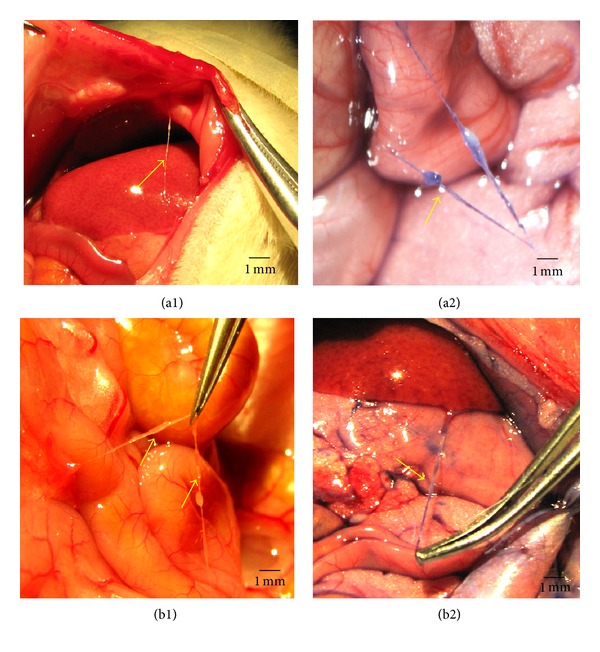
*In situ* and *in vivo* stereomicroscopic image of a typical Primo-vessel (arrow) and a corpuscle (arrow). (a1) and (a2) showed the primo vessel on the surface of intestine in the control rats. (a1) displayed that the PV was unstained and it was connected to the abnormal wall. (a2) displayed that the PV was stained with Trypan blue. (b1) and (b2) showed the primo vessel on the surface of intestine (unstained, (b1)) and stomach (stained with Trypan blue, (b2)) in PM rats. The primo vessels are semitransparent, freely movable strands irregularly fixed on the peritonea.

The PVs and the corpuscles were observed on surfaces of different internal organs such as the stomach, liver, large and small intestines, and bladder.  Figure 1 showed a representative stereomicroscopic image of a PV and its corpuscles (arrow) on the surface of the intestine. The PVs are thin, semitransparent, and freely movable strands on the peritonea, and some of them were connected to the wall of peritoneal cavity, the same as described by Lee et al. [12]. 

**Figure 2:**
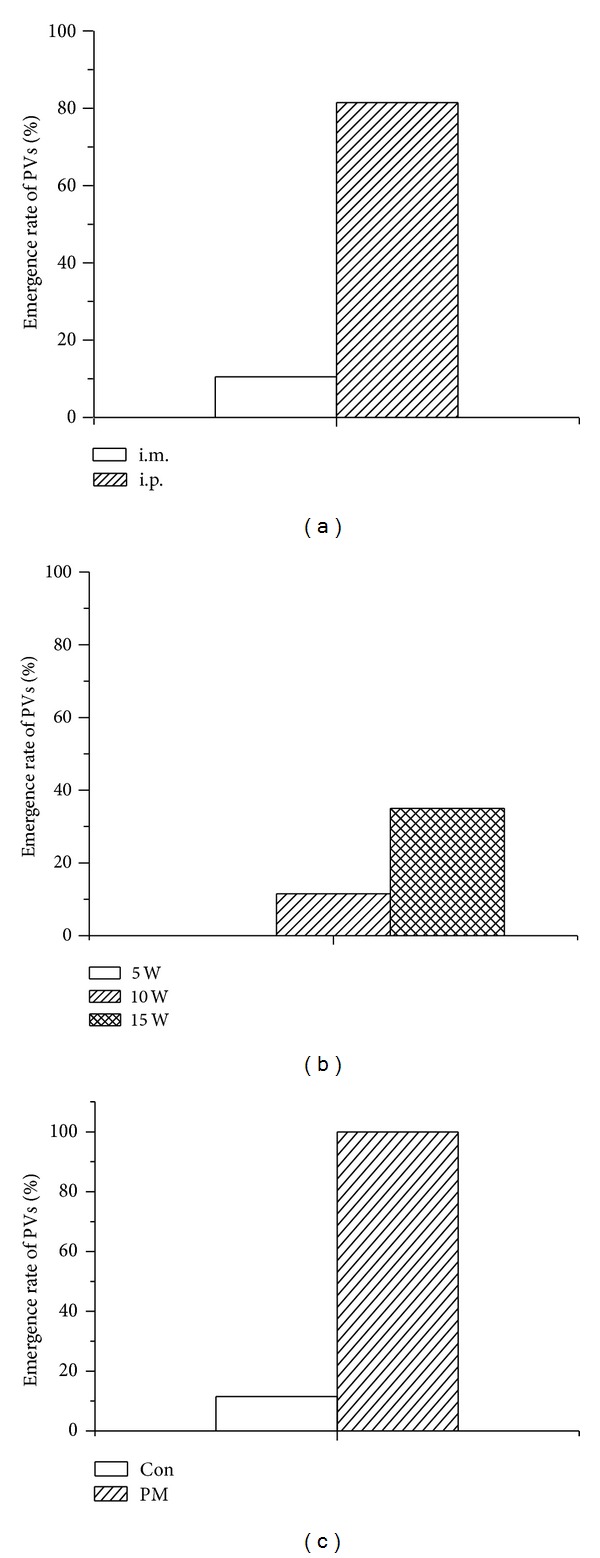
Affecting factors such as anesthesia methods, age of rats, and peritonitis on emergence rate of celiac PVs. (a) For anesthesia methods, the rate was 81.84% (22/27) with i.p. anesthetized cases which was significantly higher than 10.53% (4/38) that with i.m. (b) For the age, the rate was increased following the growth of rats in which took up 0% in 5-week old (*n* = 15) group, 10.53% (4/38) in 10-week group, and 35% (7/20) in 15-week group. (c) For the infection, the rate was 100% in the infected rats (*n* = 20), whereas the rate in the control rats was only 10.53% (4/38).

In the present study, the emergence rate of celiac PVs could be affected by urethane injection methods, age, and infection. The rate was 81.84% (22/27) with i.p. and significantly higher than that (4/38, 10.53%) in i.m. (Figure 2(a)). The rate was increased with age: 0% in 5-week old (*n* = 15) group, 10.53% (4/38) in 10-week group, and 35% (7/20) in 15-week group (Figure 2(b)). For the infection, the rate is 100% in the infected rats (*n* = 20), but only 10.53% (4/38) in the control rats (Figure 2(c)). 

### 3.2. Changes of Cytometry in Blood and Ascites of Peritonitis Model

**Figure 3:**
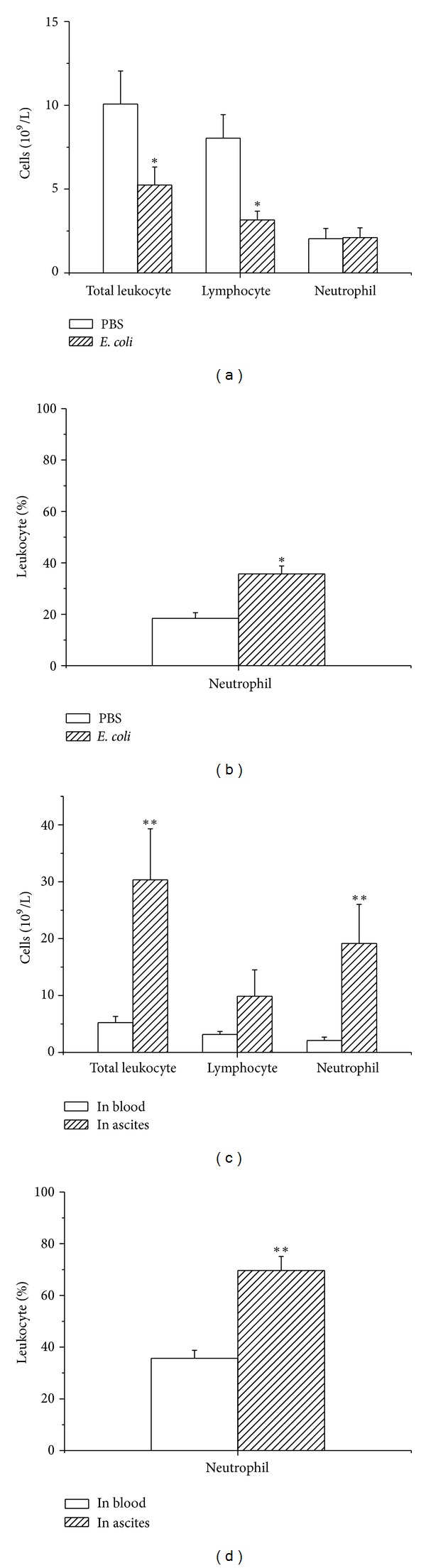
(a) and (b) showed changes of cell count in the blood 24 h after i.p. administration of *E. coli*. (a) The count of total leukocytes and lymphocytes in the blood of the PM decreased significantly (*P < 0.05) compared with the control. (b) The neutrophil increased markedly in the blood (*P < 0.05) of PM compared with that of the control. (c) and (d) showed changes of cell count in blood and ascites 24 h after i.p. administration of *E. coli*. (c) The count of total leukocyte and neutrophils in the ascites was significantly higher than that in the blood (**P < 0.01). (d) The percentage neutrophil increased significantly in the blood than that in the ascites (**P < 0.01).

After 24 h of* E. coli* injection, the rats appeared less active, less eating, and less drinking. The count of total leukocytes and lymphocytes in peripheral blood decreased significantly (*P* < 0.05). The percentage of neutrophil increased remarkably in blood (*P* < 0.05) of PM compared with that of the control (Figures 3(a) and 3(b)). The count of total leukocytes and neutrophils in the ascites was significantly higher than that in the blood (*P* < 0.01). The percentage of neutrophil increased significantly in the ascites (*P* < 0.01) than that in the blood (Figures 3(c) and 3(d)). 

### 3.3. Cell Types and Chemical Characteristics of PVs Examined with DAPI, Alexa Fluor 488 Phalloidin, CD11b, and ICAM-1

**Figure 4:**
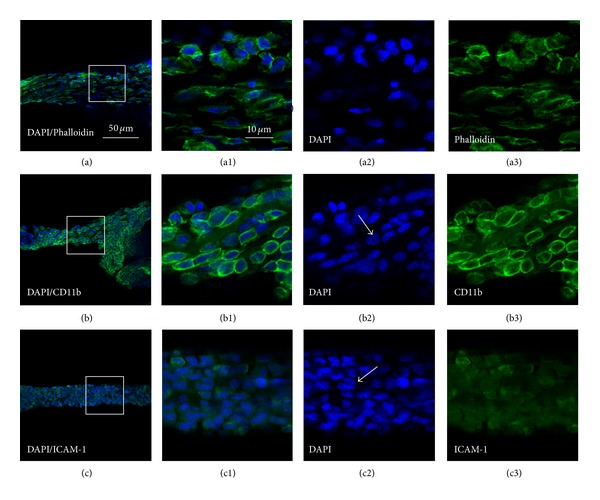
Cell types of primo vessels (PVs) in the control rats determined with DAPI/Phalloidin (a)–(a3), DAPI/CD11b (b)–(b3) and DAPI/ICAM-1 (c)–(c3) by fluorescent and immunofluorescent staining, respectively. (a)–(a3), DAPI/phalloidin-positive cells (a) and its higher resolutions (a1) emerged from images of DAPI (a2) and phalloidin (a3); (b)–(b3). DAPI/CD11b-positive cells (b) and its higher resolutions (b1) emerged from images of DAPI (b2) and CD11b (b3), typical multilobal nuclei showed in (b2) (white arrow); (c)–(c3): DAPI/ICAM-1-positive cells (c) and its higher resolutions (c1) emerged from images of DAPI (c2) and ICAM-1 (c3). Scale bar, the same for (a)–(c) (showed in (a)) and the same for the others (showed in (a1)).

**Figure 5:**
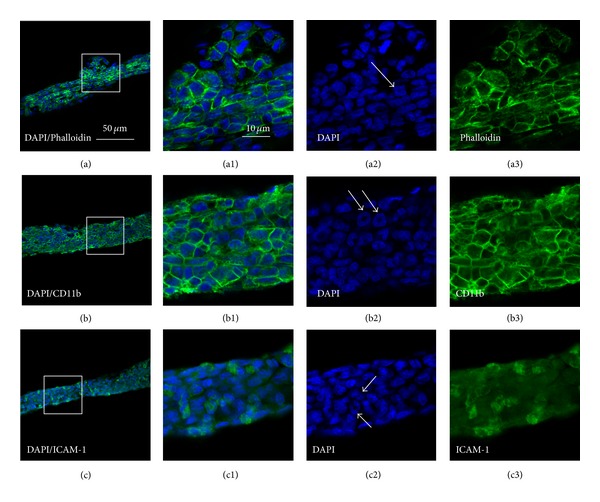
Cell types of primo vessels (PVs) in the infected rats determined with DAPI/phalloidin (a)–(a3), DAPI/CD11b (b)–(b3) and DAPI/ICAM-1 (c)–(c3) by fluorescent and immunofluorescent staining, respectively. (a)–(a3). DAPI/phalloidin-positive cells (a) and its higher resolutions (a1) emerged from images of DAPI (a2) and phalloidin (a3); (b)–(b3). DAPI/CD11b-positive cells (b) and its higher resolutions (b1) emerged from images of DAPI (b2) and CD11b (b3), typical multilobal nuclei showed in (b2) (white arrows); (c)–(c3): DAPI/ICAM-1-positive cells (c) and its higher resolutions (c1) emerged from images of DAPI (c2) and ICAM-1 (c3). Scale bar, the same for (a)–(c) (showed in (a)) and the same for the others (showed in (a1)).

The cell types and chemical characteristics of celiac PVs were examined by fluorescent staining with DAPI and Alexa Fluor 488 phalloidin, as well as immunofluorescent staining with CD11b and intercellular adhesion molecule-1(ICAM-1). We found that DAPI-, phalloidin-, CD11b-, and ICAM-1-positive labeling coexisted in PVs (Figures 4 and 5). In the PVs from control rats, the cells labeled with DAPI/phalloidin had elongated nuclei and flattened processes (Figures 4(a)–4(a3)), suggesting that fibroblasts might be an important cellular component of PVs. Using DAPI/CD11b, many CD11b-positive cells showed different shapes, and some of them closely were connected together (Figures 4(b)–4(b3)). Figures 4(c)–4(c3) showed that some of ICAM-1-positive cells are sparely distributed in PVs. In contrast, more numerous phalloidin- and CD11b-positive cells were observed in the PVs from infected rat than that from control rat (Figures 5(a)–5(a3); Figures 5(b)–5(b3). It should be noted that many CD11b-positive cells contained multilobal nucleus, showing the characteristics of neutrophil (Figures 5(b1)–5(b3)). Furthermore, there were more ICAM-1-positive cells in the infected cases than those of control cases (Figures 4(c)–4(c3); Figures 5(c)–5(c3)). 

## 4. Discussion

In this study, we found that PVs can be induced more by i.p. injection of urethane than i.m. The emergence rate of celiac PVs increased with the age. The cells of PVs in control rats included rod-shaped nuclei that aligned longitudinally along the PVs, and also CD11b and ICAM-1 were coexpressed. This indicated that some exfoliative cells or degenerative cells might contribute to the structure of celiac PVs in control and they might form a chronic inflammation. Further study showed that PVs were observed in all infected rats. DAPI-, phalloidin-, CD11b-, and ICAM-1-positive labeling also co-existed in PVs of infected rats. This indicated that high percentage of PVs emergence is due to acute peritonitis. 

In order to determine cell types and chemical characteristics in PVs, the distribution of CD11b, which is an adhesion molecule of leukocyte integrin in neutrophils surface, and ICAM-1 in celiac PVs cells were investigated. The CD11b positive cells were mainly monocytes and polymorphonuclear neutrophils and could be found in PVs. Neutrophils are the most immediate response in inflammation. CD11b directly or indirectly mediates phagocytosis and anti-infection of the neutrophils, and its increasing is also identified as the symbol during the neutrophils activation [16]. In this work, the cells of PVs in PM and controls expressed CD11b in the membrane were like neutrophils (Figures 4(b) and 5(b)). This indicated that PVs may be involved in peritonitis.

ICAM-1, as the immunoglobulin superfamily of adhesion molecules [17], could promote intercellular contact/adhesion and induce transendothelial migration of leukocytes. As a costimulatory molecule and signal transducer, it can also trigger intracellular signals and ultimately leads to the activation of lymphocytes, secretion of cytokines, and induction of proinflammatory cascades [18, 19], suggesting that ICAM-1 could be involved at the beginning of inflammation. 

Based on the present findings, fibroblasts and leukocytes might be two kinds of cell types in PVs for both of infected and untreated rats. Both CD11b and ICAM-1 were expressed in fibroblasts and leukocytes of celiac PVs. Growing evidence revealed that fibroblasts and leukocytes play an important role in early stage of inflammation. The presumed monocyte origin of fibrocytes is reflected by expression of CD11b, CD11c, and CD11d [20]. The highly expressed CD11b in the fibroblasts of PVs indicated that the activated fibroblasts might be originated from monocyte. The coexpressed CD11b and ICAM-1 of cells in PVs in peritonitis and control rats indicated that the PVs may be involved in inflammation. 

Recently studies have shown that there is a putative primo vessel inside the lymph vessel, which is proposed to participate in the cancer metastasis [21, 22]. But inflammatory process is often associated with angiogenesis and lymphangiogenesis. Studies also showed that lymphangiogenesis plays an important role in the spread of inflammation and tumor metastasis. There are considerable inflammatory cells and lymphangiogenesis existing in tumor microenvironment [23]. Thus, although Yoo et al. [13] reported that a novel function of the primo vascular system, which was different from that of lymphatics, was in conjunction with cancer events, it is easy to speculate that PVs are involved in the inflammation of tumor metastasis. 

So far, the specific function of PVs in biological processes remains unclear. As reported, the structure of the PVs is distinct from the well-known tissues such as nerves and blood vessels and may be related to acupuncture meridian [1–3]. However, our previous study demonstrated that the PVs on the surface of internal organs are involved in neither the inhibition of the gastric motility induced by acupuncturing at CV12 nor the facilitation of gastric motility induced by acupuncturing at ST36 [24]. Here our data showed that PVs were related to the process of inflammation. Further study on the role of PVs in inflammation and effect of acupuncture is encouraged in the future.

In conclusion, the emergence of PVs could be affected by age and urethane injection methods. PVs may not be an intrinsic structure of the body and may be a pathological product which is related to the process of inflammation.

## References

[1] Kim BH (1963). On the Kyungrak system. *Journal of Academy of Medical Sciences*.

[2] Kim BH (1965). Sanal theory. *Journal of Academy of Medical Sciences*.

[3] Fujiwara S, Yu SB (1967). ‘Bonghan theory’ morphological studies. *Igaku No Ayumi*.

[4] Liu X (2009). Verification and confirmation about “Bonhan ducts” as meridian. *Zhen Ci Yan Jiu*.

[5] Cho SJ, Kim BS, Park YS (2004). Threadlike structures in the aorta and coronary artery of swine. *Journal of International Society of Life Information Science*.

[6] Lee KJ, Kim S, Jung TE, Jin D, Kim DH, Kim HW (2004). Unique duct system and the corpuscle-like structures found on the surface of the liver. *Journal of International Society of Life Information Science*.

[7] Shin HS, Johng HM, Lee BC (2005). Feulgen reaction study of novel threadlike structures (Bonghan ducts) on the surfaces of mammalian organs. *Anatomical Record B*.

[8] Lee BC, Baik KY, Johng HM (2004). Acridine orange staining method to reveal the characteristic features of an intravascular threadlike structure. *Anatomical Record B*.

[9] Lee BC, Yoo JS, Baik KY, Kim KW, Soh KS (2005). Novel threadlike structures (Bonghan ducts) inside lymphatic vessels of rabbits visualized with a Janus Green B staining method. *Anatomical Record B*.

[10] Jia ZF, Lee BC, Eom KH (2010). Fluorescent nanoparticles for observing primo vascular system along sciatic nerve. *Journal of Acupuncture and Meridian Studies*.

[11] Jones JP, Bae YK (2004). Ultrasonic visualization and stimulation of classical oriental acupuncture points. *Acupuncture in Medicine*.

[12] Lee BC, Kim KW, Soh KS (2009). Visualizing the network of bonghan ducts in the omentum and peritoneum by using trypan blue. *Journal of Acupuncture and Meridian Studies*.

[13] Yoo JS, Hossein Ayati M, Kim HB, Zhang WB, Soh KS (2010). Characterization of the primo-vascular system in the abdominal cavity of lung cancer mouse model and its differences from the lymphatic system. *PLoS ONE*.

[14] Yoo JS, Kim HB, Won N (2011). Evidence for an additional metastatic route: in vivo imaging of cancer cells in the primo-vascular system around tumors and organs. *Molecular Imaging and Biology*.

[15] Wang X, Nie J, Jia Z (2010). Impaired TGF-*β* signalling enhances peritoneal inflammation induced by *E. Coli* in rats. *Nephrology Dialysis Transplantation*.

[16] Lilius E-M, Nuutila J (2012). Bacterial infections, DNA virus infections, and RNA virus infections manifest differently in neutrophil receptor expression. *The Scientific World Journal*.

[17] Staunton DE, Marlin SD, Stratowa C, Dustin ML, Springer TA (1988). Primary structure of ICAM-1 demonstrates interaction between members of the immunoglobulin and integrin supergene families. *Cell*.

[18] Salomon B, Bluestone JA (1998). LFA-1 interaction with ICAM-1 and ICAM-2 regulates Th2 cytokine production. *Journal of Immunology*.

[19] Lawson C, Wolf S (2009). ICAM-1 signaling in endothelial cells. *Pharmacological Reports*.

[20] Pilling D, Fan T, Huang D, Kaul B, Gomer RH (2009). Identification of markers that distinguish monocyte-derived fibrocytes from monocytes, macrophages, and fibroblasts. *PLoS ONE*.

[21] Lee S, Ryu Y, Lee J-K, Soh K-S, Kim S, Lim J (2012). Primo vessel inside a lymph vessel emerging from a cancer tissue. *Journal of Acupuncture and Meridian Studies*.

[22] Noh Y-I, Jung SJ, Lee S-S (2012). Isolation and morphological features of primo vessels in rabbit lymph vessels. *Journal of Acupuncture and Meridian Studies*.

[23] Alitalo K, Tammela T, Petrova TV (2005). Lymphangiogenesis in development and human disease. *Nature*.

[24] Wang X, Shi H, Shang H (2012). Are primo vessels (PVs) on the surface of gastrointestine involved in regulation of gastric motility induced by stimulating acupoints ST36 or CV12?. *Evidence-Based Complementary and Alternative Medicine*.

